# Cell-to-Cell Transformation in *Escherichia coli*: A Novel Type of Natural Transformation Involving Cell-Derived DNA and a Putative Promoting Pheromone

**DOI:** 10.1371/journal.pone.0016355

**Published:** 2011-01-20

**Authors:** Rika Etchuuya, Miki Ito, Seiko Kitano, Fukiko Shigi, Rina Sobue, Sumio Maeda

**Affiliations:** Faculty of Human Life and Environment, Nara Women's University, Nara, Japan; Universidad de la República, Uruguay

## Abstract

*Escherichia coli* is not assumed to be naturally transformable. However, several recent reports have shown that *E. coli* can express modest genetic competence in certain conditions that may arise in its environment. We have shown previously that spontaneous lateral transfer of non-conjugative plasmids occurs in a colony biofilm of mixed *E. coli* strains (a set of a donor strain harbouring a plasmid and a plasmid-free recipient strain). In this study, with high-frequency combinations of strains and a plasmid, we constructed the same lateral plasmid transfer system in liquid culture. Using this system, we demonstrated that this lateral plasmid transfer was DNase-sensitive, indicating that it is a kind of transformation in which DNase-accessible extracellular naked DNA is essential. However, this transformation did not occur with purified plasmid DNA and required a direct supply of plasmid from co-existing donor cells. Based on this feature, we have termed this transformation type as ‘cell-to-cell transformation’. Analyses using medium conditioned with the high-frequency strain revealed that this strain released a certain factor(s) that promoted cell-to-cell transformation and arrested growth of the other strains. This factor is heat-labile and protease-sensitive, and its roughly estimated molecular mass was between ∼9 kDa and ∼30 kDa, indicating that it is a polypeptide factor. Interestingly, this factor was effective even when the conditioned medium was diluted 10^–5^–10^–6^, suggesting that it acts like a pheromone with high bioactivity. Based on these results, we propose that cell-to-cell transformation is a novel natural transformation mechanism in *E. coli* that requires cell-derived DNA and is promoted by a peptide pheromone. This is the first evidence that suggests the existence of a peptide pheromone-regulated transformation mechanism in *E. coli* and in Gram-negative bacteria.

## Introduction

Lateral gene transfer between bacterial cells contributes to bacterial adaptation to various environments and, in the long term, to bacterial evolution [1]–[3]. In human environments, however, it results in the undesirable spread of pathogenic, antibiotic resistance, or artificially engineered genes [2], [4]–[8]. Three mechanisms of lateral gene transfer in bacteria are generally known: conjugation, transduction and transformation [2]. Conjugation and transduction involve specific apparatus for DNA transfer from donor cells to recipient cells; they are conjugative pili and phage capsids, respectively. However, transformation is mainly performed by the recipient cells that express genetic competence to take up extracellular free DNA [9], [10]. Competence for transformation can be induced naturally and artificially but not all bacterial species develop natural competence [1], [9], [10]. In certain Gram-positive bacteria, natural competence is induced by strain-specific competence pheromones that are secreted by a subpopulation of these bacteria [11]. Typical examples of such competence pheromones are the competence-stimulating peptide in *Streptococcus pneumoniae* [12], [13] and the ComX peptide pheromone and the competence-stimulating factor peptide in *Bacillus subtilis* [14], [15]. In contrast, definite examples of competence pheromones have not yet been reported in Gram-negative bacteria, although quorum-sensing pheromones [*N*-acyl-homoserine lactones (AHLs) and autoinducers (AIs)] possibly influence competence development indirectly [16].

*Escherichia coli* is not assumed to be naturally transformable; it develops high genetic competence only under artificial conditions, *e.g.* exposure to high Ca^2+^ concentrations [17]. However, several recent reports have shown that *E. coli* can express modest genetic competence in certain conditions that can arise in its environment [18]–[25]. Relevant to these findings, we recently found that spontaneous lateral transfer of non-conjugative plasmids occurred in an *E. coli* cell-mixed culture in a colony biofilm (a biofilm that is formed on the air–solid surface [26]–[29]) grown on common laboratory media [30] and food-based media [31]. Since non-conjugative and non-viral (or non-lysogenic) plasmids and strains were used in our experiments, we hypothesised that this plasmid transfer was due to *in situ* natural transformation in which plasmid leakage from dead cells and subsequent uptake of the free plasmid by neighbouring living cells occurred in dense colony biofilm culture [30], [31].

Here, we sought to test the ‘*in situ* transformation’ hypothesis and investigate the details of this spontaneous lateral plasmid transfer. We first demonstrated that specific combinations of strains and a plasmid that revealed high-frequency transfer in colony biofilms frequently exhibited sufficient plasmid transfer in liquid culture for use in analyses. Using such a high-frequency combination in a liquid culture system, we ascertained by DNase sensitivity whether this plasmid transfer was because of *in situ* transformation that required extracellular DNA. We next investigated whether there were any differences between this plasmid transfer and known transformation types, and the reason for the high frequency in the tested specific strain. Here, we provide data that suggest the existence of a novel transformation type in *E. coli*, termed ‘cell-to-cell transformation’, which requires cell-derived DNA and involves a putative promoting pheromone.

## Results

### Comparison of lateral plasmid transfer in colony biofilm with various combinations of *E. coli* strains and plasmids

To examine differences in the frequency of lateral plasmid transfer in colony biofilm among various strains and plasmids, we compared several combinations of *E. coli* K-12 strains and plasmids (Tables 1 and 2) [32]–[42]. Although not all combinations produced transformants, at least one combination for each donor strain (DH5, DH5α, MG1655, CAG18439, HB101 and MC4100), each recipient strain (CAG18439, HB101, MC4100 and KF1225), and each plasmid (pHSG299, pHSG399, pUC19-amp,tet and pGBM1) produced transformants, suggesting that this plasmid transfer may occur generally in *E. coli* K-12 strains and plasmids. However, several combinations containing both CAG18439 (as recipient or donor) and pHSG299 revealed very high transfer frequency between 10^–4^ and 10^–6^. This frequency was 10^6^ to 10^2^ times higher than the frequencies for other combinations (Table 2). This result suggests that specific combinations of strain and plasmid possess unknown feature(s) that strongly promote lateral plasmid transfer.

**Table 1:** *E. coli* strains and plasmids used in this study.

Strain or plasmid	Genotype or characteristics	Reference or source
**Strains**		
DH5	F^-^, *deoR, recA1, endA1, hsdR17(rK^-^, mK^+^), supE44, λ^-^, thi-1, gyrA96, relA1*	[32]
MG1655	F^-^, *λ^-^, rph-1*	[33]
HB101	F^-^, *λ^-^, hsdS20(r^-^_B_,m^-^_B_), recA13, ara-14, proA2, lacY1, galK2, rpsL20(str^r^), xyl-5, mtl-1, supE44, leu, thi*	[34]
MC4100	F^-^, *araD139, Δ(lacZYA-argF)U169, deoC1, flbB5301, ptsF25, relA1, rbsR, rpsL150(str^r^)*	[35]
CAG18439	MG1655 derivative; F^-^, *λ^-^, lacZ118(Oc), lacI3042::Tn10(tet^r^), rph-1*	[36]
KF1225	F^-^, *lacY1, galK2, tsx-29, argE3, zdd-263::Tn5(kan^r^), uidA1, mtl-1, manA4, supE44*	[37]
DH5α	F^-^, *ø80dlacZΔM15, Δ(lacZYA-argF)U169, deoR, recA1, endA1, hsdR17(r_K_^-^, m_K_^+^), phoA, supE44, λ^-^, thi-1, gyrA96, relA1*	[32]
CAG12185	MG1655 derivative; F^-^, *λ^-^, lacZ118(Oc), argE86::Tn10(tet^r^), rph-1*	[36]
CAG18475	MG1655 derivative; F^-^, *λ^-^, lacZ118(Oc), metC162::Tn10(tet^r^), rph-1*	[36]
CAG18420	MG1655 derivative; F^-^, *λ^-^, lacZ118(Oc), lacI3098::Tn10kan(kan^r^), rph-1*	[36])
AQ9950	F^-^, *araD139, Δ(lacIPOZYA)U169, rpsL, thi, lrp-201::Tn10(tet^r^)*	[38]
BW25113*ΔlacI*	Strain of the Keio Collection; F^-^, *rrnB, ΔlacZ4787, HsdR514, Δ(araBAD)567, Δ(rhaBAD)568, rph-1, ΔlacI::kan^r^*	[39]
XL1-Blue	*hsdR17*, *recA1*, *endA1*, *gyrA96*, *thi-1*, *supE44*, *relA1*,*lac* [F′, *proAB*, *LacI^q^ZΔM15*, *Tn10(tet^r^)*]	Stratagene
**Plasmids**		
pHSG299	*kan^r^*; a pUC-like high-copy cloning vector that lacks the *tra*, *mob*, and *nic-bom* regions required for conjugative transfer	[40]Accession No.: M19415
pHSG399	*cam^r^*; a pUC-like high-copy cloning vector similar to pHSG299	[40]Accession No.: M19087
pGBM1	*str^r^*; a medium-copy cloning vector containing the mutated pSC101 replicon that increases its copy number, lacking the *mob* genes and the *nic* region required for conjugative transfer	[41]
pUC19-amp,tet	Insertion of the *tet^r^* gene of pBR322 to the multicloning site in pUC19	This study
pHSG299-cam	Replacement of the *kan^r^* gene of pHSG299 to the *cam^r^* gene of pHSG399 in pHSG299	This study
pSE111	*lacI^q,^ kan^r^* and *argU* on plasmid containing p15A replicon	[42]

**Table 2:** Lateral plasmid transfer with various combinations of strains and plasmids in colony biofilm culture.

Donor cell	Plasmid	Recipient cell			
		CAG18439	HB101	MC4100	KF1225
DH5	pHSG299	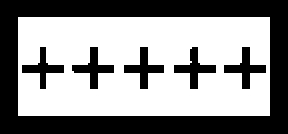	++	++	n.d.
	pHSG399	–	–	+	+
	pUC19-amp,tet	n.d.	–	–	–
	pGBM1	–	n.d.	n.d.	–
DH5α	pHSG299	++	–	–	n.d.
	pHSG399	–	–	–	–
	pUC19-amp,tet	n.d.	–	–	–
	pGBM1	–	n.d.	n.d.	++
MG1655	pHSG299	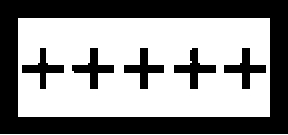	+	+	n.d.
	pHSG399	++	+++	+	++
	pUC19-amp,tet	n.d.	–	–	–
	pGBM1	+	n.d.	n.d.	–
CAG18439	pHSG299	n.d.	++	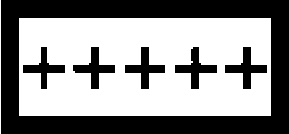	n.d.
	pHSG399	n.d.	–	–	+
	pUC19-amp,tet	n.d.	n.d.	+	n.d.
	pGBM1	n.d.	n.d.	n.d.	–
HB101	pHSG299	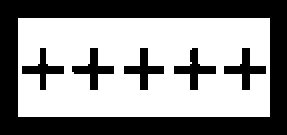	n.d.	n.d.	n.d.
	pHSG399	–	n.d.	n.d.	–
	pUC19-amp,tet	n.d.	n.d.	n.d.	–
MC4100	pHSG299	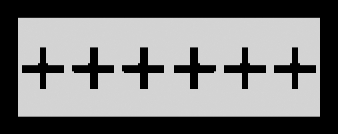	n.d.	n.d.	n.d.
	pHSG399	++	n.d.	n.d.	+
	pUC19-amp,tet	n.d.	n.d.	n.d.	–

Frequency of plasmid transfer (mean, *n* = 3) in each combination is presented in decimal ranges as follows: ++++++, 1E–4 to 1E–5; +++++, 1E–5 to 1E–6; +++, 1E–7 to 1E–8; ++, 1E–8 to 1E–9; +, 1E–9 to 1E–10; – : below detection limit; n.d., not determined or unable to examine because of coincidence of the same antibiotic resistance between strains and plasmids. Samples with plasmid transfer frequency >1E–6 are indicated by 
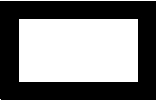
.

### Lateral plasmid transfer in liquid culture with high-frequency combinations of *E. coli* strains and a plasmid

Our previous studies revealed that lateral plasmid transfer occurred more frequently in colony biofilm cultures than in liquid cultures [30], [31]. However, colony biofilm culture is not suitable for reagent-adding analyses because it appears difficult to achieve an even diffusion of added reagents throughout the colony biofilm. Therefore, using several high-frequency combinations of strains and a plasmid listed in Table 2, we tested lateral plasmid transfer in liquid culture. We found that highly frequent plasmid transfer (∼10^–5^–10^–8^) adequate for use in analytical experiments occurred in liquid culture (Table 3). Based on these results, we adopted this cell-mixed liquid culture system for the following analyses.

**Table 3:** Lateral plasmid transfer with various combinations of strains and plasmids in liquid culture.

Donor cell	plasmid	Recipient cell	Frequency of plasmid transfer
DH5	pHSG299	CAG18439	+++++
MG1655	pHSG299	CAG18439	+++++
CAG18439	pHSG299	MC4100	+++
HB101	pHSG299	CAG18439	++++
MC4100	pHSG299	CAG18439	++++++

Frequency of plasmid transfer (mean, *n* = 3) in each combination is presented in decimal ranges as follows: ++++++, 1E–4 to 1E–5; +++++, 1E–5 to 1E–6; ++++, 1E–6 to 1E–7; +++, 1E–7 to 1E–8.

### Demonstration of transformation mechanism for lateral plasmid transfer

**Figure 1:**
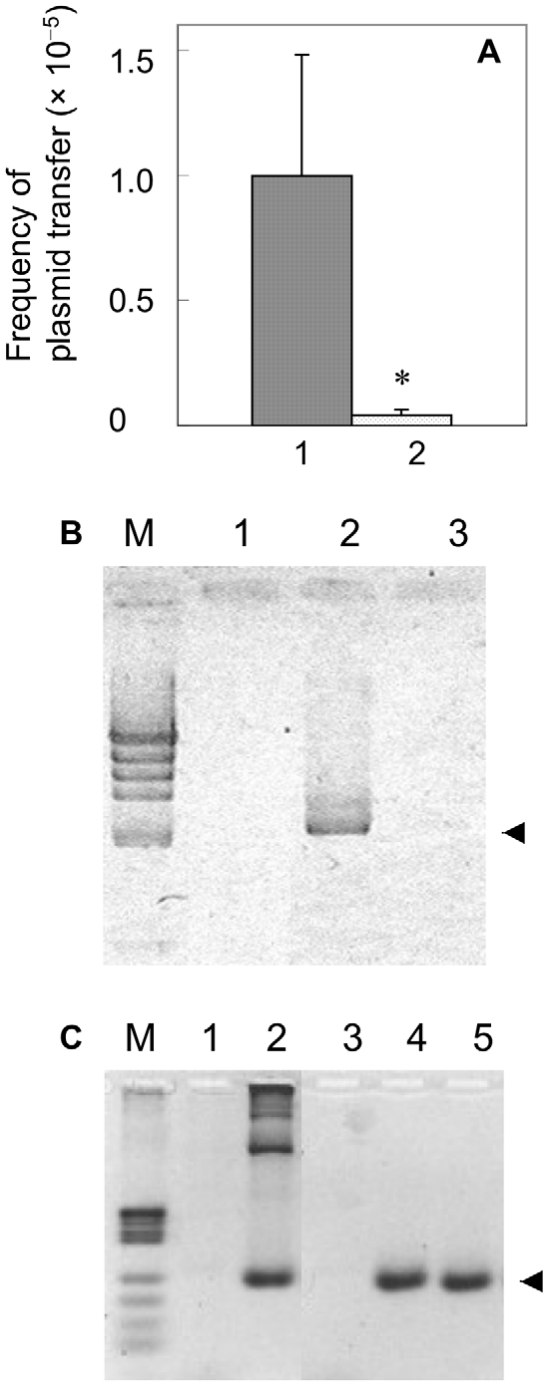
Effect of DNase I activity on lateral plasmid transfer and detection of plasmid in culture medium. Effect of DNase I activity on lateral plasmid transfer in cell-mixed culture (A, B), and detection of pHSG299 in culture medium (C). (A) Frequency of plasmid transfer [mean and standard deviation (S.D.); *: *t*-test: *P*<0.05, *n* = 5] in the absence (lane 1) and presence (lane 2) of DNase I (30 µg/mL) in a co-culture of MC4100 harbouring pHSG299 and CAG18439 in TSB. (B) Confirmation of workability of added DNase I in TSB culture. Plasmid pHSG299 DNA (10 µg/ml) and/or DNase I (30 µg/mL) was added to the co-culture of MC4100 and CAG18439 at culture start. After 16-hours culture, plasmid DNA in culture medium was isolated, digested with EcoRI and RNase A, and applied to 0.8% (w/v) agarose/Tris-borate-EDTA (TBE) gel. Lane M: size marker (lHind III); lane 1: control (no addition); lane 2: addition of purified pHSG299; lane 3: addition of purified pHSG299 and DNase I. The arrowhead shows the band of linear pHSG299 (2673 bp). (C) Detection of pHSG299 in liquid culture medium by PCR. Mixed culture medium of MC4100 harbouring pHSG299 and CAG18439 in TSB was prepared as described in Materials and methods and this medium sample was directly subjected to 0.8% (w/v) agarose/TBE gel electrophoresis (lane 1) or used as PCR template (lanes 4 and 5). Lane M: size marker (pUC119 Hpa II); lane 1: medium sample (1 µL) of MC4100 harbouring pHSG299 and CAG18439 without PCR; lane 2: positive control (PCR product from purified pHSG299 DNA); lane 3: negative control (PCR product from medium sample of plasmid-free MC4100 and CAG18439); lanes 4 and 5: PCR products from medium sample of MC4100 harbouring pHSG299 and CAG18439. The pHSG299-specific primers amplify a 229 bp fragment (arrowhead).

The most important criterion of transformation is the uptake of extracellular DNA by cells. Therefore, to ascertain whether extracellular DNA participates in this lateral plasmid transfer in our cell-mixed culture system, we examined the effect on lateral plasmid transfer of DNase I addition to the culture medium. As shown in Fig. 1A, addition of DNase I significantly decreased plasmid transfer frequency (*t*-test: *P*<0.05, *n* = 5). A control experiment confirmed that DNase I added to this culture system was active enough to degrade free DNA in the culture medium (Fig.1B). These results confirm the presence of free extracellular DNA in culture medium and its participation in plasmid transfer, and therefore demonstrate that lateral plasmid transfer in this cell-mixed culture system is due to a type of transformation mechanism.

Although the plasmid transfer experiments were carefully planned to use only non-conjugative strains and plasmids, in order to completely exclude the possibility of the involvement of conjugation we performed a filter-mediated plasmid transfer experiment (Table 4). In this experiment, recipient cells were cultured on a nylon membrane filter (pore size 0.45 µm), which was placed immediately above the plasmid-donor cells grown on agar medium. It was expected that soluble nutrients and free plasmid DNA would be able to pass through the filter, but cells and conjugative pili would be unable to pass. As shown in Table 4, conjugative transfer of F′ occurred at very high frequency (∼10^–2^–10^–3^) in mixed culture of donor and recipient cells, whereas F′ transfer was completely abolished in the filter-mediated setting. In contrast, the non-conjugative plasmid pHSG299 transferred from donor cells to recipient cells even in the filter-mediated setting. This result clearly showed that lateral plasmid transfer, as shown here, is not a type of conjugation and is not due to accidental conjugation.

**Table 4:** Lateral plasmid transfer through nylon membrane filter in colony biofilm culture.

Donor cell	plasmid	Recipient cell	Culture	Frequency of plasmid transfer
XL-1 Blue	F′	HB101	mixed	++++++++
XL-1 Blue	F′	HB101	filter-mediated	–
DH5α	pHSG299	CAG18439	mixed	++
DH5α	pHSG299	CAG18439	filter-mediated	++

Frequency of plasmid transfer (mean, *n* = 3) in each combination is presented in decimal ranges as follows: ++++++++, 1E–2 to 1E–3; ++, 1E–8 o 1E–9; – : below detection limit.

### Detection of dead cells and free plasmid DNA in cultured medium

An important premise of our hypothesis is that the transformed plasmid DNA source in cell-mixed culture is dead cells. To confirm the presence of dead cells in culture, the dead cell percentage was determined by propidium iodide (PI) staining. As given in Table 5, for example, approximately 0.8–5.7% of cells were dead at 24 hours in liquid and colony biofilm cultures. To further confirm the extracellular plasmid DNA presence, a putative free DNA fraction was purified from the liquid culture medium, which was previously centrifuged and filtered to remove cells, and analysed by agarose-gel electrophoresis. Although no plasmid DNA was detected by ethidium bromide staining in the merely purified sample (Fig. 1C, lane 1), we confirmed the presence of free plasmid DNA in the culture medium by analysing the same medium by PCR (Fig. 1C, lanes 4, 5). These results demonstrate the presence of dead cells and extracellular plasmid DNA in the culture medium.

**Table 5:** Measurement of the dead cell ratio in liquid and colony biofilm cultures.

Donor cell	plasmid	Recipient cell	Culture	Dead cell ratio* (%) (mean ± S.D., *n* = 3)
CAG18439	pHSG299	HB101	Liquid	0.8±0.9
			Colony biofilm	5.6±1.5
DH5	pHSG299	CAG18439	Liquid	1.7±0.9
			Colony biofilm	4.7±1.8

*Measured at 24 h from culture start.

### Natural transformation with purified plasmid DNA in liquid culture

To further investigate the transformation mechanism, we performed an experiment in which purified plasmid DNA was added. Using the same cell-mixed culture system as that mentioned in Table 3 except using plasmid-free strains, we examined whether added pure plasmid was transformed. As given in Table 6, no transformants were detected with any combinations of strains tested. This result indicates that purified plasmid is not used for natural transformation in liquid culture, suggesting that a specific state of plasmid DNA that is released from co-cultured donor cells is required for efficient lateral plasmid transformation in cell-mixed culture. Based on this feature, we have termed transformation in cell-mixed culture as cell-to-cell transformation, in which cell-to-cell supply of plasmid DNA during culture plays an important part. Below we use the phrase ‘cell-to-cell transformation’ in place of ‘lateral plasmid transfer’.

**Table 6:** Natural transformation with purified plasmid in liquid culture.

	Added amount of purified pHSG299	
	75 ng/mL	750 ng/mL
Strain	Transformation frequency (*n* = 3)	
CAG18439	–	–
CAG18439 (co-cultured with MC4100)	–	–
HB101	–	–
HB101 (co-cultured with DH5)	–	–
MC4100	–	–
MC4100 (co-cultured with CAG18439)	–	–

–, not detected (below detection limit).

### Comparison of cell-to-cell transformation with artificial transformation

**Figure 2:**
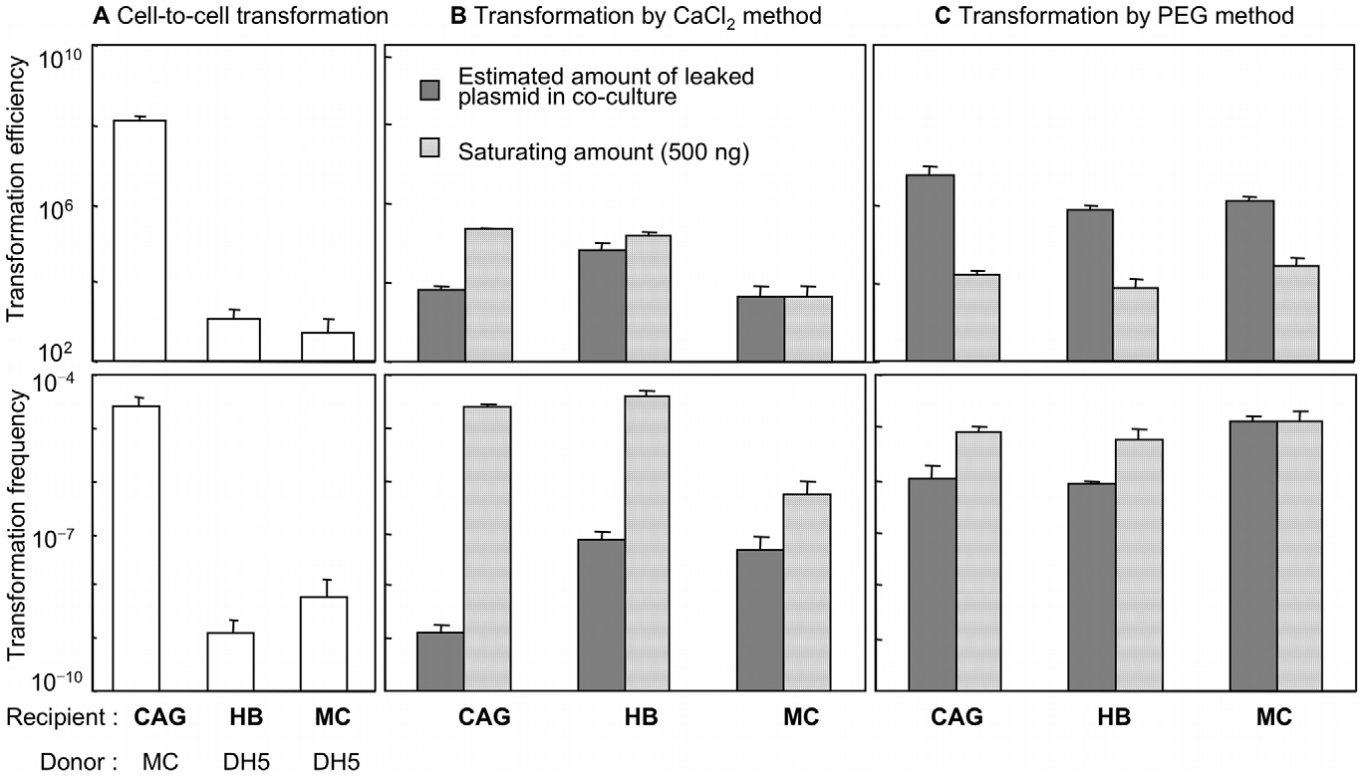
Comparison of cell-to-cell transformation with artificial transformation. Strain names: CAG, CAG18439; HB, HB101; and MC, MC4100. Cell-to-cell transformation (A) was performed with three combinations including donor cells harbouring pHSG299. Artificial transformations by the CaCl_2_ method (B) and PEG method (C) was performed as described in Methods. In artificial transformation, plasmid (pHSG299) was used in a semi-saturating amount (500 ng/sample) or the roughly estimated amount of leaked plasmid DNA in co-culture [CAG: 2×10^–10^; HB: 1×10^–8^; MC: 1×10^–9^ ng per recipient ( = competent) cells]. The latter values were calculated on the assumptions that free plasmid DNA was supplied from dead donor cells (maximum 5% of total population) to co-existing recipient cells in each corresponding co-culture of cell-to-cell transformation, and that the pHSG299 copy number is ∼200 per cell [38]. Transformation frequency and transformation efficiency were calculated as described in Methods. Data are presented as mean and S.D. (*n* = 3).

To further examine the features of cell-to-cell transformation, we compared this transformation type with conventional artificial transformation (Fig. 2). Artificial transformation was performed by the conventional CaCl_2_ or polyethylene glycol (PEG) method, and purified plasmid DNA was used in small and large amounts. The former corresponded to the roughly estimated DNA amounts that dead cells could release in co-culture and the latter was an amount estimated to be enough for semi-saturation. Figure 2 shows the results of a comparison between cell-to-cell transformation and artificial transformation using the same recipient strains. The frequency and efficiency of artificial transformation in strain CAG18439 were similar to those for other strains (HB101 and MC4100) that show a low frequency of cell-to-cell transformation (Fig. 2B, C), suggesting that the notable CAG18439 feature, which causes high-frequency cell-to-cell transformation, does not participate in artificial transformation. Therefore, it was suggested that the cell-to-cell transformation mechanism is different from that of known artificial transformation. We also found that the cell-to-cell transformation frequency and efficiency involving CAG18439 and pHSG299 were equivalent to those of artificial transformation (Fig. 2A vs. 2B, C), indicating that cell-to-cell transformation under optimal conditions is highly efficient and comparable to artificial transformation.

### Comparison of CAG18439 with other strains possessing genotypes similar to that of CAG18439

**Figure 3:**
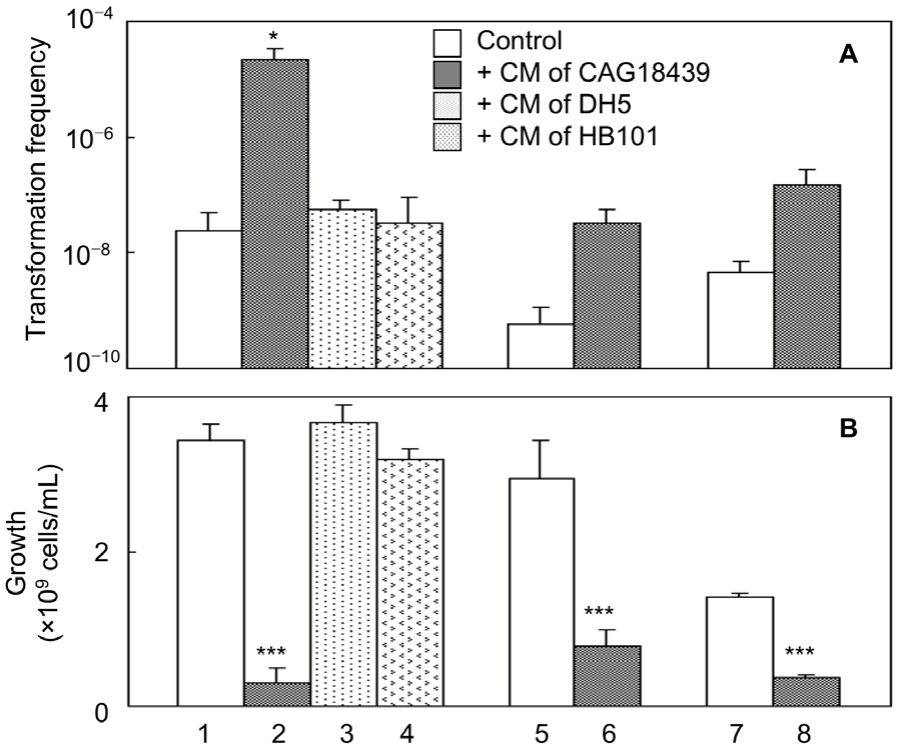
Effects of media conditioned with various strains on cell-to-cell transformation and on cell growth. Effects of media conditioned with CAG18439, DH5 and HB101 on cell-to-cell transformation (A) and on cell growth (B). Cell-to-cell transformation was performed in the presence or absence of 50% (v/v) conditioned medium (CM) of each strain indicated in the figure (lanes 1–4, co-culture of MG1655 harbouring pHSG299 and MC4100; lanes 5 and 6, co-culture of DH5 harbouring pHSG299 and MC4100; lanes 7 and 8, co-culture of MG1655 harbouring pHSG299 and MG1655 harbouring pGBM1). Data are presented as mean and S.D. (*: *t*-test: *P*<0.05, *n* = 4; †: *t*-test: *P*<0.005, *n* = 4; compared with control).

As given in Table 2, high-frequency combinations always included both CAG18439 and pHSG299. Therefore, we attempted to elucidate the mechanism of high transformation frequency in CAG18439 as follows (Table 7 and Figs. 3–6). (pHSG299 analyses are now underway and the results will be published later).

**Figure 4 pone-0016355-g004:**
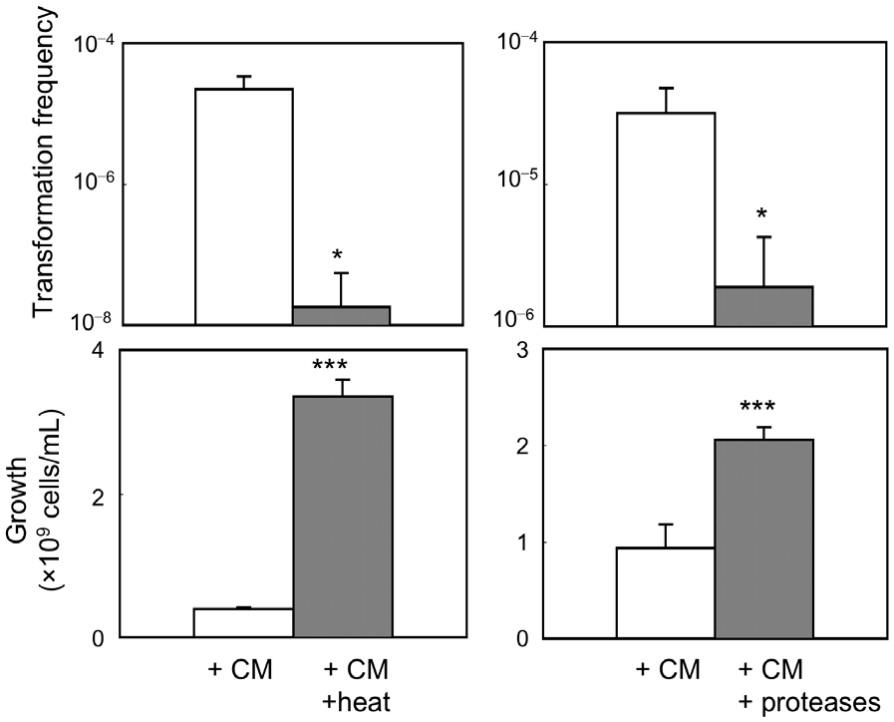
Characterization of putative active factor in CM of CAG18439. Effects of heat exposure and protease treatment of CM of CAG18439 on cell-to-cell transformation and cell growth in co-culture of MG1655 harbouring pHSG299 and MC4100. Heat treatment of CM was performed at 121°C for 20 min. Treatment of CM with trypsin (100 µg/mL) and proteinase K (200 µg/mL) was performed at 37°C for 120 min. CM was used for culture at 50% (v/v) in heat experiments and at 1% (v/v) in protease experiments. Data are presented as mean and S.D. (*: *t*-test: *P*<0.05, *n* = 4; †: *t*-test: *P*<0.005, *n* = 4).

**Figure 5 pone-0016355-g005:**
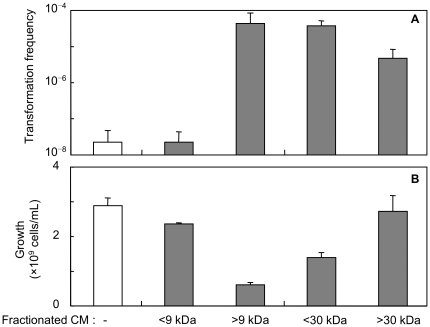
Effect of size fractionation of CM of CAG18439 on cell-to-cell transformation and cell growth. Effect of size fractionation of CM of CAG18439 on cell-to-cell transformation (A) and cell growth (B). CM was fractionated by ultrafiltration as described in Methods, and used for culture at 1% (v/v). Data are presented as mean and S.D. (*n* = 3). Co-culture of MG1655 harbouring pHSG299 and MC4100 was performed in the presence of CM.

**Figure 6:**
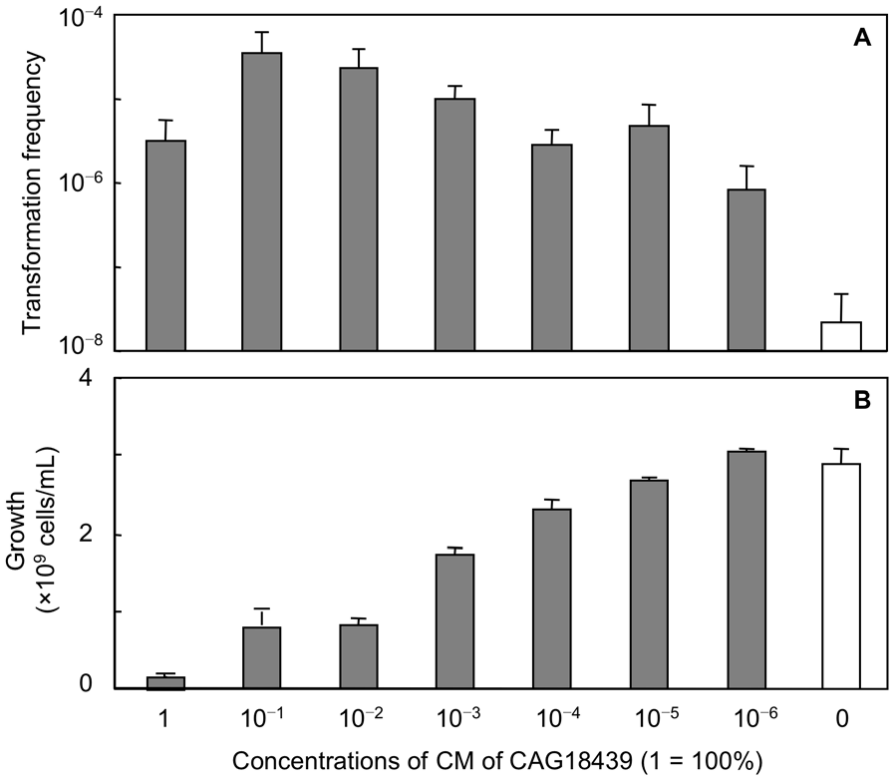
Effects of dilution of CM of CAG18439 on cell-to-cell transformation and cell growth. Effects of dilution of CM of CAG18439 on cell-to-cell transformation (A) and cell growth (B). Co-culture of MG1655 harbouring pHSG299 and MC4100 was performed in the presence of CM. Data are presented as mean and S.D. (*n* = 3).

**Table 7:** Effects of *Tn10* and *lacI* mutations on cell-to-cell transformation.

Recipient cell	Description of strain	Transformation frequency
CAG18439*	CAG strain (*lacI3042::Tn10*)	+++++
CAG12185*	CAG strain (*argE86::Tn10*)	−
CAG18475*	CAG strain (*metC162::Tn10*)	−
CAG18420†	CAG strain (*lacI3098::Tn10kan*)	−
AQ9950*	*Δ(lacIPOZYA)U169*	−
BW25113*ΔlacI* †	*ΔlacI*	+
CAG18439 harboring pSE111†	*lacI^q^* on plasmid	++++

DH5 harbouring pHSG299 (*) or DH5 harbouring pHSG299-cam (†) was used as the donor strain. Transformation frequency (mean, *n* = 3) in each combination is presented in decimal ranges as follows: +++++, 1E−5 to 1E−6; ++++, 1E−6 to 1E−7; +, 1E−9 to 1E−10; −, below detection limit.

The CAG strains are MG1655 mutant series including a *Tn10* insertion in the chromosome, established by Singer *et al*. [36]. Therefore, the activities of other CAG strains as recipient cells were examined (Table 7). However, the other CAG strains tested did not show a high-frequency transformation, suggesting that high frequency is not due to the genetic features (including *Tn10*) commonly present in the CAG strains. In particular, the lack of activity in CAG18420, which is also a *lacI::Tn10* derivative of the CAG strains, indicates that neither *Tn10* nor *lacI* mutation is the cause of high activity in CAG18439. Consistent with this suggestion, neither other *lacI* mutants nor plasmids carrying the *lacI^q^* gene revealed apparent effects (Table 7). These results suggest that unidentified mutation(s) in the CAG18439 chromosome other than *Tn10* and *lacI* cause the high transformation frequency in CAG18439.

### Effect of CAG18439-conditioned medium on cell-to-cell transformation between strains other than CAG18439

In high-frequency combinations, it was observed that CAG18439 caused high transformation frequency not only as the recipient cell but also as the donor cell (Tables 2 and 3). This suggests that the key function of CAG18439 to promote cell-to cell transformation is not directly related to its own recipient or donor functions. It is possible that CAG18439 produces and secretes an unknown factor that acts on co-existing cells in a paracrine (and perhaps also autocrine) manner and promotes cell-to-cell transformation. We therefore examined the effect of CAG18439-conditioned medium (50%, v/v) on cell-to-cell transformation between strains other than CAG18439. Interestingly, the CAG18439-conditioned medium promoted cell-to-cell transformation (×∼1000–30) in some combinations of other strains (Fig. 3A). This effect was large (×∼1000) and significant (*t*-test: *P*<0.05, *n* = 4) in the combination of MG1655 harboring pHSG299 and MC4100. However, conditioned medium of other low-frequency strains (DH5 and HB101) did not show such an effect (Fig. 3A). Similar promoting effects were also observed when CAG18439 was present as a third strain in a mixed culture of two other strains (data not shown).

Moreover, when we measured donor cells/recipient cells ratio in cell-mixed culture experiments, we found that co-culture with CAG18439 resulted in repressed growth of the counter strain in many combinations, *i.e*. CAG18439 was in majority (∼80–99%) in cell-mixed culture. Therefore, we also examined the effect of CAG18439-conditioned medium on the growth of other strains (Fig. 3B). CAG18439-conditioned medium actually revealed growth arrest activity [×∼1/12–1/4, (*t*-test: *P*<0.005, *n* = 4)] (Fig. 3B), while medium conditioned with other strains did not show such activity (Fig. 3B). The growth arrest activity was not the result of cell killing because dead cells (by PI stained) did not increase on addition of the conditioned medium (data not shown). These results suggest that CAG18439 produces and releases a soluble factor that can promote cell-to-cell transformation and arrest growth of other *E. coli* cells.

### Effect of heat treatment, protease digestion and size fractionation of medium conditioned with CAG18439 on cell-to-cell transformation

To examine whether the soluble factor in medium conditioned with CAG18439 is a protein or peptide, we performed the following three experiments. As shown in Fig. 4, exposure of CAG18439-conditioned medium to heat (121°C, 20 min) or treatment of the conditioned medium with proteases (proteinase K and trypsin) resulted in significant decrease in the ability of the medium to promote cell-to-cell transformation (*t*-test: *P*<0.05, *n* = 4, for both treatments) and growth arrest (*t*-test: *P*<0.005, *n* = 4, for both treatments). Furthermore, size fractionation (Fig. 5) of the CAG18439-conditioned medium by ultrafiltration revealed that the factor may be >∼9 kDa and <∼30 kDa. Although the activity was also present in the non-passage fraction of >∼30 kDa, this can be explained by adsorption of the factor to the ultrafiltration membrane or retained residues. These results suggest that the factor responsible for both promotion of cell-to-cell transformation and growth arrest is a protein or a polypeptide.

### Effect of dilution of CAG18439 conditioned medium

The minimum active concentration of CAG18439-conditioned medium was examined by diluting this medium. Surprisingly, the medium was effective up to the dilutions of 10^–5^–10^–6^ in promoting cell-to-cell transformation (Fig. 6A). Growth arrest activity also occurred at low concentrations but it was weaker and almost lost at the dilution of 10^–5^ (Fig. 6B). These results suggest that the factor present in CAG18439-conditioned medium acts as a bioactive signal factor like a pheromone, which can transduce specific signals from certain cells to other cell populations at extremely low concentrations.

## Discussion

From the above results, we drew the following two conclusions: (1) spontaneous lateral plasmid transfer in mixed *E. coli* co-culture systems results from cell-to-cell transformation occurrence, which requires cell-derived DNA and is performed through a mechanism that differs from simple natural transformation and artificial transformation; (2) a specific *E. coli* strain (CAG18439) produces and releases peptide pheromone-like factor(s) that can act at very low concentrations (10^–5^–10^–6^ times dilution of conditioned medium) to promote cell-to-cell transformation and to arrest growth of the other strains.

The occurrence of cell-to-cell transformation was deduced from the following three lines of evidence: (1) lateral plasmid transfer was decreased by degrading extracellular DNA in culture with DNase I treatment (Fig. 1A, B), demonstrating the transformation mechanism of this lateral plasmid transfer; (2) the presence of extracellular plasmid DNA in the medium and dead cells as its possible source (Fig. 1C, Table 5), supporting a transformation mechanism in which transformed DNA is supplied in culture *in situ*; and (3) failure to take up the purified plasmid (Table 6), suggesting the requirement for a cell-to-cell supply of plasmid DNA for this transformation to occur.

Although natural transformation in *E. coli* with artificially added purified DNA was reported by several researchers including us, our results reported previously [30], [31] and the present results provide the first evidence demonstrating that spontaneous cell-to-cell natural transformation occurs in *E. coli* co-culture systems without the artificial addition of purified plasmid DNA or any special treatments.

Despite the growing numbers of examples of natural transformation in *E. coli*, its mechanism is largely unknown. At the culture level, several reports [20], [24] including ours [23], [30], [31] suggested that natural transformation in *E. coli* occurs more frequently in solid cultures than in liquid cultures. This study showed for the first time that natural transformation in *E. coli* can occur in liquid culture at high frequency if several specific conditions are provided. Therefore, it was revealed that solid culture is not essential for efficient natural transformation in *E. coli*.

Several results suggest the requirement for trace amounts (<0.5 mM) of Ca^2+^ (and Mg^2+^) for natural transformation in *E. coli* [18], [43], similar to artificial transformation. However, some results cannot be explained solely based on the action of such ions [19], [22], [23], [25], [31]. The results of this study also show that, despite using the same medium, transformation frequency varies extremely (maximum ∼10^6^ times the difference) according to the strain and plasmid combinations and the presence of the putative promoting pheromone. Therefore, we think that trace amounts of Ca^2+^ and Mg^2+^ ions may be required for the DNA-uptake mechanism but are not the dominant factors.

At the genetic level, recently, the *com* gene homologues, which are reported to be involved in natural transformation in other Gram-negative bacteria [10], were also found in *E. coli*. Finkel & Kolter [44] proposed that in *E. coli* these *com*-like gene products act mainly in the starved culture condition, in which DNA is the sole carbon source. However, our results were obtained under nutrient-rich conditions and we therefore think that the involvement of *com*-like genes in cell-to-cell transformation is unlikely. The type IV secretion system (T4SS) is also known to be involved in natural transformation in Gram-negative bacteria [10], [45], [46]. However, the *E. coli* strains used in this study did not contain any conjugative plasmids containing T4SS genes [47]. Therefore, the involvement of T4SS in cell-to-cell transformation is unlikely.

Besides, as shown in Fig. 2, the abilities of strains to perform cell-to-cell transformation as the recipient cells did not correlate with their abilities to perform artificial transformations. Therefore, we postulate that cell-to-cell transformation may occur through a mechanism different from artificial transformations. It is possible that natural transformation in *E. coli* may have several variations of pathways. This possibility has also been proposed by Sun *et al*. [43].

We postulated that plasmid DNA was probably supplied naturally from dead cells through cell disruption because this is the most natural manner of DNA supply in culture. Consistent with this idea, a small amount of extracellular plasmid DNA and dead cells was detected in the culture medium (Table 5 and Fig. 1C). We performed a preliminary test of the ability of dead cells as the source of transformed DNA, and found that the plasmid DNA included in dead cells could be transformed by cells in culture, at least in colony biofilm culture (data not shown). However, since artificial cell-killing manipulations often damage DNA and proteins, a clear demonstration of the involvement of dead cell DNA would require further carefully planned experiments.

Regarding the requirement for cell-derived DNA for cell-to-cell transformation to occur, the results of Tsen *et al*. [20] seem to be partly related to the results of this study. They reported that natural transformation in *E. coli* on agar media was promoted by the co-presence of added plasmid and *E. coli* cell lysate, which was obtained by *in situ* mild disruption of the cells with ampicillin treatment. Their result suggests that *E. coli* lysate promotes plasmid uptake, and this appears relevant to our result that DNA derived from co-cultured cells is required for cell-to-cell transformation. A variety of materials can be released from dead cells, and some of them (such as DNA-binding proteins and lipopolysaccharide) have the abilities to associate with DNA [48], [49]. The preferred transfer of pHSG299 (Table 2) may suggest the presence of specific sequence(s) that promotes cell-to-cell transformation by binding of such DNA-associating molecule(s). Similar transformation-promoting sequences has been reported as DUS (DNA uptake sequences) or USS (uptake signal sequences) in other Gram-negative bacteria [10], [50]–[52]. Alternatively or additionally, a DNA conformation specific to living cells, such as supercoiling, may also be a requirement for the substrate of cell-to-cell transformation.

DNA secretion as a physiological process of living cells has been proposed in several bacterial systems [53]. This may be another possible DNA-supply mechanism in cell-to-cell transformation. Presently, we have no additional evidence to clarify the mechanism of DNA release from donor cells or the role of dead or living donor cells. Including these points, the detailed molecular mechanism of cell-to-cell transformation should be investigated further.

In this study, we also suggested for the first time the presence of a novel pheromone-like factor that promotes cell-to-cell natural transformation in *E. coli*. Bacterial pheromones such as AHLs are generally known to work at nM concentrations [54]. Although presently we do not know the exact concentration of the promoting factor in our experimental system, our detection of this activity in conditioned medium diluted to 10^–6^ (Fig. 6) is consistent with the concept of a pheromone with high biological activity. Our data on heat sensitivity, estimated molecular mass and protease sensitivity (Figs. 4 and 5) consistently indicate that the putative pheromone consists of a polypeptide. No peptide factors showing similar activity have been identified in *E. coli* or other Gram-negative bacteria [10]. Because of the presence of an outer membrane in Gram-negative bacteria, it is believed that polypeptide-type factors cannot transmit signals easily from the outside of cells. However, a few reports postulate the presence of peptide pheromones in Gram-negative bacteria [55], [56]. In Gram-positive bacteria, several competence factors or pheromones were found to be peptide factors [12]–[15]. These data support an idea that a peptide-type competence pheromone may also be present in *E. coli*. However, we cannot conclude that the factor we found is a ‘competence’ pheromone because we did not clarify whether the factor acts on donor cells or recipient cells. If the effect is on donor cells, the factor may promote the release of plasmid DNA from donor cells. In other bacteria, there is a phenomenon termed ‘fratricide’, in which a competence pheromone also has cell-killing activity that leads to the release of DNA from other cells [57]. However, since we did not find a cell-killing activity for the putative factor, such a scenario is unlikely to be involved in cell-to-cell transformation. Alternatively, in donor cells the factor may up-regulate unknown DNA-associating molecule(s) that can promote uptake by recipient cells when they are released together with plasmid DNA. This idea seems to be consistent with our finding of a requirement for cell-derived DNA in cell-to-cell transformation.

Besides, we found growth arrest activity in medium conditioned with CAG18439 (Fig. 3). Although this activity and the activity promoting cell-to-cell transformation behaved similarly toward physical and biochemical treatments (Figs. 4–6), whether this activity is involved in cell-to-cell transformation is unclear at present. The transformation-promoting activity appears to be effective on CAG18439 itself because CAG18439 can exhibit high activity as both donor and recipient (Tables 2 and 3). However, the growth-arrest activity appears to be ineffective on CAG18439 itself because CAG18439 growth in sole culture is apparently normal. Therefore, these two effects may act on cells independently. The target(s), the ranges of actions and the working mechanisms of this putative pheromone are to be investigated further.

CAG18439 is a *Tn10*-insertion derivative of MG1655, established by Singer *et al*. [36]. Based on the results in Table 7, unidentified mutation(s) in CAG18439 may cause expression of pheromone activity. Our preliminary study in progress suggests that a few other *E. coli* K-12 strains may have a similar activity promoting cell-to-cell transformation. Therefore, production of this pheromone may not be specific to CAG18439. The identity and the gene for this pheromone as well as the responsible mutation in the CAG18439 chromosome should be investigated.

It is noteworthy that, under optimal conditions, cell-to-cell transformation occurred as frequently as artificial transformations (Fig. 2). This means that cell-to-cell transformation in *E. coli* (perhaps also between or in other bacteria) may occur in environments outside the laboratory at non-negligible frequencies if several conditions are met. In this respect, further experiments using natural strains of *E. coli* and other bacteria will be required. Furthermore to our results, other recent results [18]–[20], [24], [58] suggest that non-conjugative plasmids are more mobile than was previously believed. Reevaluation of plasmid dynamics in various environments is needed to confirm this possibility [4]–[6].

## Materials and Methods

### *E. coli* strains, plasmids, and materials

The *E. coli* strains and plasmids used in this study are listed in Table 1. The following *E. coli* strains and plasmids were obtained from the “National BioResource Project (NIG, Japan): *E. coli*” (http://www.shigen.nig.ac.jp/ecoli/strain/top/top.jsp): DH5, MG1655, HB101, MC4100, CAG18439, CAG12185, CAG18475, CAG18420, AQ9950, BW25113*ΔlacI*, KF1225, pHSG299, pHSG399, pUC19, pGBM1, and pSE111. Plasmid pUC19-amp,tet was constructed by insertion of the pBR322 *tet^r^* gene into the multi-cloning site in pUC19. Plasmid pHSG299-cam was constructed by replacement of the pHSG299 *kan^r^* gene with the pHSG399 *cam^r^* gene in pHSG299 by PCR. Strain DH5α, XL1-Blue and PCR enzyme KOD Dash were obtained from Toyobo. Ampicillin (amp), tetracycline (tet), streptomycin (str), chloramphenicol (cam), PI, PEG (molecular mass  = 8000) and LB powder (Luria-Bertani, Lennox) were from Sigma. Tryptic Soy Broth (TSB) was from Becton, Dickinson. Distilled water (DNase- and RNase-free, molecular biology grade) and kanamycin (kan) were from Invitrogen. Nylon66-membrane filter (pore size: 0.45 µm, Biodyne A) was from Pall. DNase I (bovine pancreas, Grade II) was from Boehringer Mannheim. Syringe filters for sterilisation (pore size: 0.20 µm) were from Iwaki. Agar powder (guaranteed-reagent grade), proteinase K, trypsin, and other general reagents were from Wako.

### Plasmid transfer experiments in colony biofilm

Lateral plasmid transfer experiments in a colony-biofilm system were performed as described previously [30], [31]. However, the protocol was modified slightly based on tentative data from studies of the experimental conditions. Briefly, the plasmid-harbouring donor cells (possessing an antibiotic resistance gene on the plasmid) and recipient cells (possessing another antibiotic resistance gene on the chromosome or on another compatible plasmid) were pre-cultured separately in antibiotic-free LB broth or broth containing suitable antibiotics (75 µg/mL kanamycin, 75 µg/mL tetracycline, 75 µg/mL streptomycin, or 100 µg/mL chloramphenicol) at 37°C for 7 hours with shaking, washed once with fresh PBS (1.47 mM potassium phosphate monobasic, 8.1 mM sodium phosphate bibasic, 2.7 mM potassium chloride and 137 mM sodium chloride, pH 7.4 at 25°C), and mixed at a 1∶1 ratio (2×10^9^ cells/mL each). Aliquots (20 µL; total 8×10^7^ cells) were then spotted onto pieces of sterilised nylon membrane filter (20×20 mm) and cultured on antibiotic-free TSB agar (1.5% w/v) at 37°C for 16 hours. The colony biofilms that formed were collected and spread onto LB agar plates containing two antibiotics to select recipient cells that had acquired plasmids. The occurrence of lateral plasmid transfer was detected by the appearance of double-resistant transformants. Transformation frequency was calculated as the ratio of the transformant number to the estimated recipient cell number, which was regarded as half of the total cell number in each sample. The total cell number in each sample was deduced from the OD_600_ value of the cell suspension just before plating.

### Plasmid transfer experiments in liquid culture

Lateral plasmid transfer experiments in liquid culture were performed by following the same protocol as that for colony biofilm experiments, except that cell-mixed culture was performed in 1 mL TSB with shaking. In experiments using liquid culture, for more precise calculation of plasmid transfer frequency, the donor cells/recipient cells ratio after cell-mixed culture was determined as follows. The cell mixture after culture was diluted and plated on antibiotic-free LB agar. The colonies produced were streaked individually (∼200 per sample) on fresh LB agar containing the antibiotic to which the recipient cells are resistant. The resulting sensitive cells/resistant cells ratio was regarded as the donor cells/recipient cells ratio. This ratio was used in calculating the recipient cell number; the resultant value was used for calculation of plasmid-transfer frequency described above. Exceptionally, in the case of co-culture of MG1655 harbouring pHSG299 and MG1655 harbouring pGBM1, the total cell number was regarded as the recipient cell number, because all the cells can act as recipient cells.

### Filter-mediated plasmid transfer experiments in colony biofilm culture

Filter-mediated plasmid transfer experiments in colony biofilm culture were performed using a protocol similar to that used for simple colony biofilm experiments. Donor cells containing F′ or pHSG299 were cultured as described above. Nylon membrane filter (20×20 mm) was placed immediately above the colony biofilm of donor cells, and recipient cells were inoculated onto the filter and cultured at 37°C for an additional 24 hours. The recipient cells were then recovered from the filter and plated on LB agar plates containing two antibiotics to select recipient cells that had acquired plasmids.

### Measurement of dead cells by microscopy with PI staining

Cell solutions were prepared with PBS at a concentration of 1×10^8^ cells/mL. Stock solution of PI (100 µg/mL) was added to this cell solution at 1∶1 (v/v) and was incubated at 25°C for 5 min. Dead cell numbers, which were stained with PI, and total cell numbers were counted by phase-contrast microscopy and fluorescent microscopy (excitation, 485 nm; emission, 520 nm), respectively.

### PCR analysis

To detect pHSG299 DNA in the culture medium, PCR was performed using primers for pHSG299, as described previously [30], [31]. Cultured medium samples (each 1 mL) were centrifuged and filtered using the same protocol as that used for conditioned medium described below. Five µL of this filterd medium was mixed with 5 µL of the Chelex solution (Bio-Rad), heated at 99°C for 5 min, and briefly centrifuged (10000 *g*, 10 min). One µL of this supernatant was subjected to PCR.

### Natural transformation experiments

Natural transformation experiments in liquid culture were performed as follows: *E. coli* cells were pre-cultured, cultured and plated using essentially the same protocol as that used for lateral plasmid transfer experiments, except that plasmid DNA (75 ng or 750 ng), which was purified by an alkali method and phenol-chloroform extraction, was added to the medium (1 mL) at the culture start.

### Artificial transformation experiments

The CaCl_2_ method was performed by a conventional protocol using 100 mM CaCl_2_ [32], [59]. The PEG method was performed as described by Chung *et al*. [60]. Transformation frequency was calculated as the ratio of the transformant number to the recipient (competent) cell number. Transformation efficiency (per µg DNA) was calculated as the ratio of the transformant number to the added amount of plasmid DNA.

### Preparation and ultrafiltration of conditioned medium

Conditioned medium was prepared as follows. Cells were grown in TSB at 37°C for 7 hours with shaking. This culture solution was centrifuged (5000 *g*, 10 min), and the supernatant was filtered with a membrane filter (pore size: 0.20 µm) to remove residual cells completely. For size fractionation of the conditioned medium by ultrafiltration, the prepared conditioned medium was centrifuged in Amicon Ultra-4 (3 K) (7500 *g*, 30 min) or Microcon Ultracel YM-10 (10 K) (14000 *g*, 30 min). According to the manufacturer's information, molecule sizes in flow-through fractions were regarded as 3-times larger than those described on the product labels. Therefore, the estimated molecular mass of the molecules in the flow-through fractions with 3 K and 10 K filters was regarded as <9 kDa and <30 kDa, respectively. After centrifugation, the ultrafiltration membrane was washed onece with fresh TSB, and the residual materials retained on the membrane was withdrawn by pipetting with fresh TSB. The resultant solutions were regarded as >9 kDa and >30 kDa fractions, respectively.
